# Meat Quality and Sensory Traits of Finisher Broiler Chickens Fed with Black Soldier Fly (*Hermetia Illucens* L.) Larvae Fat as Alternative Fat Source

**DOI:** 10.3390/ani9040140

**Published:** 2019-04-02

**Authors:** Marco Cullere, Achille Schiavone, Sihem Dabbou, Laura Gasco, Antonella Dalle Zotte

**Affiliations:** 1 Department of Animal Medicine, Production and Health, University of Padova, Agripolis, Viale dell’Università 16, 35020 Legnaro, Padova, Italy; Antonella.dallezotte@unipd.it; 2 Department of Veterinary Sciences, University of Turin, Largo Paolo Braccini 2, 10095 Grugliasco (TO), Italy; achille.schiavone@unito.it (A.S.); sihem.dabbou@unito.it (S.D.); 3 Department of Agricultural, Forest and Food Sciences, University of Turin, Largo Paolo Braccini 2, 10095 Grugliasco (TO), Italy; laura.gasco@unito.it; 4 Institute of Science of Food Production, National Research Council, Largo Paolo Braccini 2, 10095 Grugliasco (TO), Italy

**Keywords:** *Hermetia illucens*, animal feed, alternative feed source, sustainability, dietary fat source, broiler chickens, meat quality, fatty acids, sensory traits

## Abstract

**Simple Summary:**

Insects represent a promising feed ingredient for poultry diets, as an alternative to the conventional feedstuffs. Once the black soldier fly (*Hermetia illucens*; HI) larvae are collected, they are processed to obtain two main products: the protein and the fat fractions. Currently, the possible utilization of the fat fraction in poultry diets has only partly been investigated, providing encouraging results in terms of chickens’ performance, health status, intestinal morphology and histological features. However, its effect on meat quality, which is extremely important from the consumer’s point of view, has scarcely been investigated yet. Therefore, the present research studied the effect of 0%, 50% and 100% dietary replacement of soybean oil with HI larvae fat on the quality and sensory characteristics of chicken meat. Overall results were comparable among the three dietary groups, suggesting that HI larvae fat can be considered a promising sustainable ingredient for poultry diets which can feasibly be used for feed formulations. However, the fat composition of the larvae is sub-optimal for providing healthy meat for the modern consumer. For this reason, further research to solve this drawback is needed.

**Abstract:**

At present, there is limited knowledge about the possible utilization of the fat fraction derived from *Hermetia illucens* (HI) larvae processing. The objective of the present study was to evaluate the replacement of soybean oil with HI larvae fat in broiler finisher diet, on the quality and sensory traits of their meat. At 21 days of age, 120 male broiler chickens were randomly allocated to three experimental groups (5 replicates and 8 birds/pen): a basal control diet (C), and two groups in which either 50% or 100% of the soybean oil was replaced with HI larvae fat (the HI50 and HI100 group, respectively). At day 48, 15 birds (3 birds/pen) per group were slaughtered, and breasts and legs were excised and used for meat quality evaluations. Breast and leg physical meat quality, nutritional composition and sensory profile remained substantially unaffected by the dietary treatments. In contrast, the dietary incorporation of HI larvae fat modified the fatty acid (FA) profile of both the breast and leg meat cuts: the proportion of saturated fatty acids increased (*p* < 0.0001) to the detriment of the polyunsaturated (PUFA) fraction (*p* < 0.0001). Moreover, the meat *n*-6/*n*-3 ratio increased in the HI50 and HI100 groups compared to the C group. HI larvae fat dietary inclusion decreased the monounsaturated fatty acids in the breast (*p* = 0.0012) but not in the leg meat. Further research should focus on the improvement of the FA profile of the larvae through substrate modulation, or by combining HI larvae with a PUFA-rich feedstuff in feed formulations.

## 1. Introduction

Starting from the position paper of the Food and Agriculture Organization of the United Nations [1] which pointed out the urgent need to find alternative feed sources to satisfy an increasing demand for animal products due to the rising world population, a growing number of studies testing the potential of different insect species as feed for different animals have been published. Among the insect species that have been gaining attention as alternatives to the conventional feedstuffs for poultry, the black soldier fly (*Hermetia illucens* L.; HI) is certainly one of the most promising, due to its intrinsic nutritional value, the possibility of exploiting diverse substrates for farming and because it is suitable for mass production [2].

Up to now, defatted HI meal has been the feed ingredient which has been the most appealing for the feed industry because it is very rich in protein [3], thus making it a possible substitute for soybean meal. Consequently, research efforts have also been mostly directed towards this emerging feed ingredient, studying the optimal defatted HI meal incorporation levels intended for different poultry species of economic interest. Dietary inclusions ranging between 5% and 19% have been tested in broiler chickens [3,4], broiler quails (*Coturnix coturnix japonica*) [5,6], ducks [7] and Barbary partridges (*Alectoris barbara*) [8,9]. Findings in all these avian species were encouraging, as general health status, growth performance, carcass and meat quality traits, were satisfactory and suggested a possible practical application of the defatted HI meal in the formulation of poultry diets.

Partially or highly defatted protein meal, however, is only one of the products that can be obtained from the HI larvae. In fact, the industrial processing of this insect species to produce the protein meal, generates another co-product which is quantitatively (30%–40% of the dry matter (DM)) and nutritionally relevant: the fat fraction. The fat derived from HI larvae was studied as a possible source for biodiesel production and provided interesting results [10], but it could also possibly be exploited in the formulation of poultry diets as a substitution for the commonly used lipid sources such as rendered fat and vegetable oils, in particular that obtained from soybean. This is because HI larvae fat is rich in medium chain fatty acids (MCFA) that can have positive effects on gut health, thus improving growth performance [11]. Moreover, MCFA was shown to be preferentially utilized as an energy source, improving energy availability without increasing the deposition of lipids [12]. Despite its potential, there is still a limited number of research papers dealing with the dietary application of HI larvae fat in meat-producing birds. HI larvae fat has been included as a partial (50%) or total (100%) replacement of soybean oil in the diet for broiler chickens from one day of age up to slaughter (35 days) [13]. Independently of the replacement percentage, it was observed that chickens fed with HI larvae fat had satisfactory productive performance, carcass traits and overall meat quality. The only possible drawback was the fatty acid (FA) profile of the meat which was negatively influenced by the new ingredient: the total saturated fatty acids (SFA) increased to the detriment of the polyunsaturated fatty acid (PUFA) fraction. Based on these initial results, it was decided to test the same fat source on the same bird species, but limiting the supplementation period solely to the finisher phase, when dietary SFA are used to improve the firmness of the carcass fat depots. The first part of this work considered growth performance, carcass traits, blood parameters, intestinal morphology and histological features [14] and no adverse effects were observed for the studied traits. Therefore, the present research was aimed at evaluating the effects of a partial (50%) or total (100%) replacement of soybean oil with HI larvae fat in finisher broiler chickens’ diets on the t physical traits, proximate composition, cholesterol content, oxidative status, fatty acid profile and sensory traits of the meat.

## 2. Materials and Methods

The present experiment (experimental protocol no. 380576, December 4, 2017) was approved by the Bioethical Committee of the University of Turin (Italy).

### 2.1. Experimental Design

For the present experiment, three dietary treatments were tested on chicken broilers (ROSS 708) from 21 to 48 days of age (finisher diet): a basal control diet (C: 100% soybean oil), and two diets with either 50% or 100% replacement of soybean oil with HI larvae fat (HI50 and HI100 groups, respectively). The diet that was fed to day-old chicks up to 21 days of age was a commercial broiler starter diet containing 217 g/kg crude protein and 12.9 MJ/kg metabolizable energy. Detailed information about the chemical composition of the diets, chicken farming conditions and live performance are provided in the study by Schiavone et al. [14].

A total of 45 chickens from 15 birds/experimental group (3 birds/pen, 5 pens/dietary treatment) were considered for meat quality evaluations. From each slaughtered bird, breasts and legs were excised and analyzed at the Laboratory of the Department of Animal Medicine, Production and Health, MAPS, of the University of Padova (Italy). Each breast was halved, and each half breast was labeled, vacuum-packaged using a CSV-41n ORVED machine (99% vacuum level) in polyethylene bags (water vapor transmission rate: 3.5 ± 1 g/m^2^ day at 23 °C and 85 ± 2% relative humidity) and stored at −40 °C until analysis. The same procedure was adopted for the right and left leg of each chicken. Overall, a total of *n* = 45 right breasts, *n* = 45 left breasts, *n* = 45 right legs and *n* = 45 left legs were assigned to meat quality evaluations.

### 2.2. Physical Meat Quality

After 2 weeks of frozen storage, all right breasts and legs were weighed, allowed to thaw for 12 h at 4 °C and weighed again to calculate the thawing loss (%). The pH was measured in the cranial and caudal part of the *Pectoralis major* muscle for the breast, and in the *Iliotibialis lateralis* muscle for the leg (portable pH meter FG2-Five GoTM; Mettler Toledo, Greifensee, Switzerland; calibration at pH 4.0 and 7.0). Color measurements were performed on the *Iliotibialis lateralis* muscle (RM200QC colorimeter; X-Rite Co., Neu-Isenburg, Germany) and considered lightness (L*), redness (a*) and yellowness (b*) [15]. pH as well as the color values represented the average of two repeated measurements. Legs were then vacuum-sealed using the above-mentioned equipment and cooked in a water bath set at 80 °C, until the core temperature reached 77 °C. Then, samples were cooled in an ice bath, dried and weighed to calculate the cooking and total losses (%).

### 2.3. Chemical Meat Quality

After physical evaluations, all right breasts were ground with a Retsch Grindomix GM 200 (7000 g for 10 s); an aliquot of about 20 g/breast was dedicated to the thiobarbituric acid-reactive substances (TBARs) analysis, whereas the remaining meat/breast was frozen at −40 °C, freeze-dried and ground again (7000 g for 5 s) to obtain a fine powder which was used to determine: proximate composition, FA profile and cholesterol content. The left legs were thawed for 12 h at 4 °C, deboned and then they were processed following the same procedure described for breast meat samples. The same meat quality evaluations conducted on the right breasts, were performed on the left legs.

The proximate composition of breast and leg meat samples was analyzed in accordance with the AOAC [16] methods. The cholesterol content was determined through absolute quantitative analysis using HPLC following the method described by Casiraghi et al. [17]. The extent of muscle lipid oxidation was evaluated with a spectrophotometer (Hitachi U-2000; Hitachi, Mannheim, Germany) set at 532 nm, that measured the absorbance of TBARs and a 1,1,3,3-tetraethoxypropane calibration curve [18]. Oxidation products were quantified as malondialdehyde (MDA) equivalents (mg MDA/kg muscle).

### 2.4. Fatty Acid Profile of Hermetia illucens Fat, Soybean Oil, Diets and Meat Samples

The lipid extraction was performed by Accelerated Solvent Extraction (M-ASE) using different solvents according to the specific matrix: petroleum ether for HI larvae fat, soybean oil and diets, and chloroform:methanol 1:2 for breast and leg meat samples. The FA profile was determined as described by Cullere et al. [6]. Samples were transmethylated using a methanolic solution of H2SO4 (4%) in order to determine fatty acid methyl esters (FAME). A biphasic separation was obtained by adding 0.5 mL of distilled water and 1.5 mL of N-heptane to each sample. FAME were quantified by gas chromatography (Shimadzu GC17A), equipped with an Omegawax 250 column (30 m × 0.25 µm × 0.25 µm) and Flame Ionization Detector. Helium was used as the carrier gas at a constant flow of 0.8 mL/min. The injector and detector temperatures were 260 °C. Peaks were identified based on commercially available FAME mixtures (37-Component FAME Mix, Supelco Inc., Bellefonte, PA, USA). The results are expressed as the percentage (%) of total detected FAME. The FA composition of the *Hermetia ilucens* fat, soybean oil and experimental diets is presented in Table 1.

### 2.5. Sensory Analysis

Left breast meat samples were subjected to a descriptive sensory analysis, to detect possible differences among the dietary treatments (C vs. HI50 vs. HI100). The sensory analysis was performed by an eight-member trained panel (Istituto per la Qualità e le Tecnologie Agroalimentari, Laboratorio Analisi Sensoriale—Veneto Agricoltura, Thiene, Vicenza, Italy), whose members were qualified as experts according to ISO 8586 and had experience with descriptive tests (ISO 13299) on various food matrices. All judges who perform tests with accredited methods undergo training every three years. Panelists underwent two pre-test training sessions of 1 h each to familiarize them with the matrix and select appropriate descriptors, also drawn from the literature. The panel received a list of descriptors to score on numerical and continuous scales from 0 (the lowest score for each attribute) to 10 (the highest score for each attribute). Olfactory, gustative and textural aspects were evaluated. The descriptors were: chicken odor, chicken flavor, saltiness, unctuosity, acidity, fibrousness, chewiness, juiciness, adhesiveness and toughness. The meat used for the training sessions was that of chicken breasts fed with a conventional diet which was processed, stored, handled and cooked in the same manner of the samples which were used for the subsequent sensory analysis.

For the experiment, a total of 15 chicken breasts/treatment were used and 2 days of analysis were scheduled (7 and 8 breasts/treatment/session). After 1 month of frozen storage at −40 °C, chicken breasts were allowed to thaw for 16 h at 4 °C. Each sample was then placed on a cooking plate (model GR6010 XL Health Comfort, 2400 Watt; Rowenta, Erbach, Germany) set at thermostat position ‘2’ and cooked 8 min/side, until the core temperature reached 74 °C. Subsequently, samples were put in aluminum trays and served to the panel in a random sequence. Samples were identified by a random three-digit code. The evaluation sheet, distribution of samples to the judges and data acquisition were performed using FIZZ software package 2.60.4(BIOSYSTEMES FRANCE, St-Ouen l’Aumône, France) installed in eight terminals in the tasting booths of the laboratory. Still water at room temperature and unsalted crackers were available to panelists throughout each sensory session.

### 2.6. Statistical Analysis

Data were subjected to a one-way ANOVA with experimental diet (C, HI50 and HI100) as fixed effect, following the GLM procedure of the SAS 9.1.3 Statistical Analysis Software for Windows [19]. A mixed model (PROC MIXED) was used to detect any dietary influence on sensory analysis scores, therefore considering experimental diet and the eight panelists as fixed and random effects, respectively. Least square means were obtained using the Bonferroni test and the significance was calculated at a 5% confidence level.

## 3. Results

Chicken breast derived from birds fed with diets containing 0%, 50% or 100% substitution of soybean oil with HI larvae fat, overall showed similar physico-chemical meat quality traits (Table 2). Specifically, breasts displayed similar weight, thawing loss and pH (*p* > 0.05). Also, the proximate composition did not differ among the three experimental groups which showed comparable water, protein, lipids and ash contents. An increasing replacement of soybean oil with HI larvae fat did not affect the oxidative status of the meat as chicken breasts displayed similar TBARs values in the three groups. In contrast, the cholesterol content of the HI50 chicken breasts was significantly lower compared to the C and HI100 groups (59.4 vs. 64.2 and 66.9 mg/100 g meat for the HI50, C and HI100 groups, respectively; *p* = 0.0003). This result was not expected since in the HI50 group the HI larvae fat, a cholesterol-rich feedstuff (412.8 mg cholesterol/100 g fat), substituted for only 50% of the soybean oil, whereas the 100% substitution level did not provide the same outcome.

As was observed for the breast meat cut, the physical traits of the chicken leg were also not affected by the experimental diets including different incorporation levels of HI larvae fat (Table 3). In fact, leg weight, thawing and cooking losses, pH, and L* (lightness) a* (redness) b* (yellowness) color values displayed similar values in the three experimental groups.

Leg meat nutritional quality was not affected by the increasing substitution of soybean oil with HI larvae fat (Table 4). Also, cholesterol content, in contrast to what was observed for breast meat, did not differ among the experimental groups (80.2 vs. 79.7 vs. 81.5 mg/100 g meat, for the C, HI50 and HI100 groups, respectively).

The dietary substitution of soybean oil with HI larvae fat significantly changed the fatty acid (FA) profile of the chicken breast meat (Table 5). Most of the saturated FA (SFA) increased with growing HI larvae fat inclusion levels, and the most affected were lauric acid (C12:0; *p* < 0.0001), myristic acid (C14:0; *p* < 0.0001) and palmitic acid (C16:0; *p* = 0.0004). Different from most SFA, C20:0 decreased with the increasing the HI larvae fat inclusion level (*p* = 0.0002). As a result, the total SFA incidence significantly (*p* < 0.0001) differed among treatments (28.8 vs. 36.5 vs. 45.8% of total FAME for C, HI50 and HI100, respectively).

Different from what was observed for SFA, the monounsaturated FA (MUFA) fraction evidenced an inverse trend when substituting the soybean oil with the HI larvae fat. Despite some minor MUFA increases from the C to the HI100 groups (C14:1 and C16:1; *p* < 0.0001), C20:1 *n*-9 and, particularly, C18:1 *n*-9 (oleic acid) consistently decreased (*p* < 0.0001) which determined the following MUFA proportions in the three groups: 27.7 < 25.6 < 23.5 5 total FAME for the C, HI50 and HI100 chicken breasts (*p* = 0.0012).

A similar situation to that observed for the MUFA, but even more remarkable, was obtained for the polyunsaturated FA (PUFA) fraction, which decreased with increasing substitution levels of soybean oil with HI larvae fat: 36.8 vs. 30.6 vs. 23.4% total FAME for the C, HI50 and HI100 dietary groups, respectively (*p* < 0.0001). This change was mainly due to the C18:2 *n*-6 (linoleic acid; *p* < 0.0001) and the C18:3 *n*-3 (α-linolenic acid; *p* < 0.0001). Consequently, the substitution of soybean oil with HI larvae fat significantly (*p* < 0.0001) increased the *n*-6/*n*-3 ratio: 15.8 vs. 18.0 vs. 21.7 for the C, HI50 and HI100 dietary groups, respectively (*p* < 0.0001). Due to the extensive changes in the FA profile of chicken breast meat, the atherogenic index (AI), thrombogenic index (TI) and hypocholesterolemic/hypercholesterolemic (HH) ratios decreased, thus worsening from the C to the HI100 groups (*p* < 0.0001). Opposite to this, the peroxidability index (PI) decreased in the following manner: 57.3 vs. 51.3 vs. 42.4 for the C, HI50 and HI100 dietary groups, respectively (*p* < 0.0001).

As observed for breast meat, the FA profile of the chicken leg was also significantly affected by the dietary treatments and a similar trend was observed (Table 6). The SFA content increased mainly due to the lauric, myristic and palmitic FA (*p* < 0.01). Conversely, even though C17:0, C18:0 and C20:0 followed the opposite trend (*p* < 0.0001), their relative contributions were insufficient to counteract the growing pattern of the other SFA (SFA: 28.8 vs. 36.5 vs. 45.8% total FAME for the C, HI50 and HI100 dietary groups, respectively; *p* < 0.0001). Despite the oleic acid decrease with the increasing substitution level of soybean oil with HI larvae fat (*p* < 0.001), the overall MUFA proportion remained unchanged in the three dietary groups (28.9, 27.5 and 26.1% total FAME for the C, HI50 and HI100 dietary groups, respectively). As observed for the breast meat cut, in the leg meat the PUFA fraction also decreased when chickens’ diets contained HI larvae fat (*p* < 0.0001), and the *n*-6/*n*-3 ratio increased (*p* < 0.0001). This result was mainly attributable to the decrease of linoleic and arachidonic (C20:4 *n*-6) FAs, and α-linolenic FA. Consequently, health indexes of FA showed the same trend previously mentioned for the breast, worsening with the increasing HI larvae fat inclusion level.

Overall, considering the FA profile results of both chicken breast and leg meat, it can be stated that the replacement of soybean oil with HI larvae fat consistently changed the FA profile of chicken breast meat, generally lowering its healthiness for human consumption.

As for the sensory traits of the chicken breasts, selected descriptors such as chicken odor, flavor, saltiness, fibrousness, chewiness, juiciness, adhesiveness, toughness, unctuosity and acidity were similar in the C, HI50 and HI100 groups (Table 7).

## 4. Discussion

The fat fraction of the HI larvae represents a promising ingredient quantitatively and nutritionally, with possible application in livestock feeding which could alleviate the pressure on conventional overexploited feed sources. For this reason, in some recent research the dietary incorporation of HI larvae fat in juvenile Jian carp [12], rainbow trout [20] and rabbit [21,22,23] was studied. Regardless of the animal species, results provided positive outcomes which were consistent with those of the present research, as the overall nutritional composition of the studied animal products remained unaffected by the insect fat-based dietary treatments. Recent findings highlighted that HI larvae can lower lipid deposition in the intraperitoneal fat tissue of Jian carp due to an augmented lipid hydrolysis mediated through the mRNA up-regulation of PPARα, a gene involved in FA synthesis, which decreased adipocytes’ size [12]. Additionally, lauric acid (C12:0) was shown to lower fat deposition in rabbits as it is oxidized to CO_2_ more rapidly than other long-chain FA, thus being less available to be stored in tissues [22]. However, a similar lowering effect on the lipid deposition related to feeding HI larvae fat to finisher chickens was not observed in the present study, as the lipid content of the breast and leg meat cuts were similar in the three dietary treatments.

The present study confirmed the richness of the HI larvae fat in cholesterol, which was previously also observed in the study by Caligiani et al. [24] where the cholesterol content ranged from 1.2 to 1.5 g/100 g fat. Despite this, increasing the HI larvae fat dietary inclusion in chickens’ diet did not augment the cholesterol content of the chicken leg meat as was expected. On the contrary, it is difficult to explain why the breast meat of the HI50 group contained the lowest amount of cholesterol, whereas the extreme dietary treatments (C and HI100) showed the highest.

Cholesterol is known for being an essential element in vertebrate cell membranes and an obligatory precursor of steroid hormones, bile acids, and oxysterols [25]. In different animal species, including birds, increases in dietary cholesterol levels are compensated for by changes in lipid metabolism in the liver, including decreased de novo cholesterol synthesis and/or increased transformation and transportation. These are essential to preserve health status, welfare and productivity [26,27]. For these reasons, dietary changes should affect meat cholesterol content only to a moderate extent. In the literature, it has been observed that the dietary presence of ingredients containing phytosterols in chickens’ diet lowered the cholesterol content of the skeletal muscle as a possible result of the blocked reabsorption of bile acids and cholesterol in the ileum by phytosterols [28]. However, this was not confirmed in the present study, as chicken fed with 100% soybean oil produced breast meat with the same cholesterol content as that obtained from HI100 chickens. Also, animal growth rate was found to be inversely correlated with the cholesterol content of both chicken breast and thigh muscles [28]. However, in the first part of the present study [14] chickens in the three dietary treatments displayed similar daily growth rates and final live weights, thus making it not possible to relate growth to meat cholesterol content. Up to now, no other study has observed a meat cholesterol-lowering effect possibly attributable to an insect-based feed product. The only available results deal with laying poultry species. In laying quails fed with defatted HI larvae meal (10% or 15% inclusion levels), eggs did not display a reduction in their cholesterol content [29] consistent with the fact that most animal species, including birds, have tight cholesterol regulation. In contrast, Secci et al. [30] found that laying hens fed with HI larvae meal as a 100% replacement for soybean meal showed the lowest cholesterol content in the egg yolk. The authors attributed this finding to a reduction in the serum cholesterol level, caused by the chitin content, which should attract negatively-charged bile acids and free FA. However, the latter mechanism cannot explain the present findings in chicken meat as HI larvae fat does not contain chitin.

The observed enrichment of the breast and leg chicken meat in medium-chain FA with increasing incorporation levels of HI poses, on the one hand, an issue regarding meat healthiness as lauric (C12:0) and myristic (C14:0) FA are well known for their hypercholesterolemic properties [31]. However, it was also demonstrated that diets rich in lauric acid, which was the second most abundant SFA in chicken meat in the present study, have a greater proportional effect on high-density lipoprotein (HDL) than on low-density lipoprotein (LDL) levels, thus lowering the total cholesterol/HDL cholesterol level [32]. It is also true that the above-mentioned FAs are also physiologically active compounds which are preferentially utilized as an energy source which has been shown to improve energy availability without increasing the deposition of lipids [12]. The only other published work dealing with the dietary inclusion of HI larvae fat in chickens’ diets as a replacement for soybean oil is that of Schiavone et al. [13] which, however, tested experimental diets from the starter to the finisher phases, thus for the chickens’ whole production cycle. The present and the above-mentioned study showed that, independent of the duration of the dietary treatment, the resulting effects on overall meat quality traits were similar: physical meat quality remained unaffected, as well as the proximate composition of the meat.

The results on the FA profile of both the breast and leg chicken meat in the present experiment highlighted that 27 days duration of dietary treatment was enough to generate relevant effects on the FA profile of the meat. In the present trial, the addition of HI larvae fat into chickens’ diets increased the SFA content of the breast meat to a higher extent than was observed in the work by Schiavone et al. [13] (17.4% and 35.1% increase in HI50 and HI100 diets, respectively, compared to the control group). Moreover, the decreasing pattern in the breast MUFA proportion, from the control to the HI100 groups, was not observed in the study of Schiavone et al. [13], where the breast MUFA content remained unaffected by increasing the dietary incorporation of HI larvae fat. Fatty acid deposition is the result of absorption, de novo synthesis, and β-oxidation of FA [33]: elongase and Δ-9 desaturase (stearoyl-CoA desaturase) enzyme activities from C12:0, C14:0 and C16:0 FA, whose dietary content augmented with increasing incorporation level of HI larvae fat, might have compensated for the lower dietary intake of MUFA [34]. However, as the addition of a double bond is a rate-limiting step in MUFA biosynthesis rather than the simple chain elongation, it was observed that elongation activity (insertion of two carbon units at the carboxyl terminal of FAs) tends to be higher than desaturation (addition of a double bond). Furthermore, on the one hand, surplus amounts of SFA were shown to block the biosynthesis of some MUFA, especially 18:1 *n*-9, by suppressing elongase activity [35]. On the other hand, dietary PUFA was shown to reduce Δ-9 desaturase enzymatic activity, thus lowering the conversion of SFA into MUFA [33]. Therefore, the delicate equilibrium between dietary FA absorption and the contribution of dietary FA to modulate enzymatic activity could explain the results of the present research.

The observed reduction of both the dietary *n*-6 and the *n*-3 proportions of the PUFA fraction when increasing HI fat levels directly reflected that of the meat. The reduction was more intense for the *n*-3 than for *n*-6 FAs, which led to the highest *n*-6/*n*-3 ratio. This pattern found confirmation in the existing literature on chickens [13] but also on rabbits [22]. Up to now, the research conducted on the use of HI larvae in livestock farming has highlighted that modification of the FA profile of the diets, and thus meat, towards higher saturation of lipids was an issue not limited to the use of the fat fraction of the HI larvae, but also extended to its protein meal. In fact, the dietary inclusion of higher levels of defatted HI protein meal in rainbow trout [36], quail [6] and Barbary partridge [9] diets led to significant modifications in the FA profile of their meat, worsening its healthiness overall by increasing and reducing the SFA and the PUFA contents, respectively, as well as increasing the PUFA *n*-6/*n*-3 ratio.

Such FA modifications were constant in different animal species, meat cuts, and different dietary inclusion levels of the studied innovative ingredients, thus highlighting the need to adopt appropriate strategies to cope with this issue. Firstly, it was recently demonstrated that the killing procedure for HI larvae can have a great effect on the quality of their lipid fraction. In fact, HI larvae have a high concentration of the lipase enzyme [37] which is activated when the larvae are subjected to direct freezing as the killing procedure. This generates selective hydrolytic reactions, with a further selective cleavage of unsaturated FA by lipase enzymes and a subsequent intense saturation of the larvae FA profile [24]. Despite this, also when lipase enzyme was inactivated by thermal treatment, SFA were lower but were still the predominant FA fraction of HI larvae lipids. Secondly, the specific FA composition of the larvae can be also affected by the rearing substrate, even if to a limited extent: independent of the FA composition of the feed, HI larvae demonstrated they preferentially synthesized lauric acid mainly from dietary carbohydrates, which is deposited in large amounts in their body [38]. Despite the physiological role of lauric acid in insects not yet being characterized, it has been demonstrated to be involved in insect development, particularly during the transition from larval to adult life, and during adult maturation [39], as well as in the production of antimicrobial and antiviral compounds [40], thus explaining why, independent of the growing medium for HI larvae, it is always the main FA. The limited effect of the rearing substrate on the HI larvae FA composition was also further demonstrated when HI larvae were fed in a substrate enriched with fish offal, as it was observed that their FA composition was mainly constituted of SFA (72% of total FAME), as is commonly observed in this insect species reared on any other substrates, whereas the PUFA content decreased (6.99% of total FAME). The *n*-3 fraction and the *n*-6/*n*-3 ratio benefited from the fish offal inclusion, but it was not enough to improve the overall healthiness of the lipids [41]. Similarly, HI larvae reared in a substrate enriched with *n*-3 PUFA (50% *Ascophyllum nodosum* and 50% processed wheat), increased their content of *n*-6 and *n*-3 PUFA to 25.3%, but SFA accounted for more than the 60% of the total FAME. Unfortunately, the substrate enrichment with *n*-3 PUFA had a drawback in ensuring satisfactory productive performance of larvae [42]. Based on such considerations, it seems therefore that the easiest and most effective way to improve the quality of the lipids in a feed formulation containing either HI larvae fat or protein meal would be to include another feed ingredient of vegetable origin rich in PUFA and in the *n*-3 fraction, such as linseed [43].

The breast meat of chickens fed with increasing levels of HI larvae fat showed a similar sensory profile to the meat of those fed with a conventional soybean oil-based diet. This finding, which is very important for the consumer point of view in accepting the use of this alternative ingredient in animal feed, is supported by previous research considering the sensory profile of meat derived from different animal species fed with HI protein meal. In fact, the sensory traits of quail breasts derived from birds fed either with 10% and 15% defatted HI meal were comparable to those obtained from quails fed with a conventional soybean meal diet [6]. In fish, no or only small changes were detected in the fillet sensory quality when HI meal was included in the diet [44,45,46].

The HI larvae meal were recently reported to have a distinctive flavor, described as earthy, chocolate/malt with delicate fish notes, and the fat was characterized by even more pronounced chocolate/malt flavors than the meal [47]. These descriptors were however not used in the present study, as the first aim was to focus on the typical descriptors used to characterize chicken meat.

## 5. Conclusions

The present research showed that the replacement of soybean oil with *Hermetia illucens* larvae fat in the diet for finisher broiler chicken is technically feasible, up to the 100% substitution level. Moreover, overall chicken meat quality was satisfactory from a nutritional and sensory point of view, thus suggesting that the *Hermetia illucens* larvae fat can be considered a possible ingredient to be included in commercial diets for broiler chickens. The fatty acids profile of the derived meat, however, is the sole drawback related to the use of this alternative feed ingredient as it was negatively enriched in saturated fatty acids, mainly to the detriment of the polyunsaturated fatty acid fraction. To solve this problem, two possible strategies can be adopted: improve the fat composition of larvae through substrate modulation or equilibrate the lipid quality of the poultry diet by including, in the feed formulation, ingredients rich in polyunsaturated fatty acids, especially of the *n*-3 series, in combination with the *Hermetia illucens* larvae fat.

## Figures and Tables

**Table 1:** Cholesterol content of the *Hermetia illucens* (HI) larvae fat and fatty acid profile (% of total FAME) of the HI larvae fat, soybean oil and experimental diets with 0% (Control, C), 50% (HI50) and 100% (HI00) substitution of soybean oil with HI larvae fat.

	Fat Sources		Experimental Diets		
	Soybean Oil	HI Larvae Fat	C	HI50	HI100
Cholesterol, mg/100 g	-	413	-	-	-
*Fatty acid composition:*					
C12:0 (Lauric acid)	0.00	52.6	0.00	17.8	34.7
C14:0 (Myristic acid)	0.09	8.54	0.00	3.38	6.50
C16:0 (Palmitic acid)	11.5	10.9	13.2	12.6	13.6
C18:0 (Stearic acid)	3.31	1.53	2.82	2.41	2.12
Total SFA	14.9	75.0	16.0	36.2	56.9
C14:1	0.00	0.17	0.00	0.00	0.00
C16:1	0.00	1.98	0.00	0.90	1.72
C18:1 *n*-9 (Oleic acid)	23.1	6.16	22.4	18.0	12.9
C18:1 *n*-11	1.51	0.24	1.41	1.03	0.57
Total MUFA	24.7	8.55	23.8	19.9	15.2
C18:2 *n*-6 (Linoleic acid)	53.8	11.6	54.9	40.1	26.2
C18:3 *n*-3 (α-Linolenic acid)	5.94	1.01	4.88	3.22	1.66
C20:4 *n*-6 (Arachidonic acid)	0.00	0.29	0.00	0.00	0.00
Total PUFA	59.7	12.9	59.8	43.3	27.9
UFA/SFA	5.65	0.29	5.22	1.75	0.76
*n*-6	53.8	11.9	54.9	40.1	26.2
*n*-3	5.94	1.01	4.88	3.22	1.66
*n*-6/*n*-3	9.10	11.8	11.2	12.5	15.8
Identified FA (%)	99.3	96.5	99.6	99.4	100.0

FAME = fatty acid methyl esters; MUFA = monounsaturated fatty acids; PUFA = polyunsaturated fatty acids; SFA = saturated fatty acids; UFA = unsaturated fatty acids; FA = fatty acid.

**Table 2:** Physico-chemical quality of the breast meat of chickens fed with 0% (C), 50% (HI50) and 100% (HI100) substitution of soybean oil with *Hermetia illucens* (HI) larvae fat in their finisher diets.

Physico-Chemical Traits	Experimental Groups			*p*-Value	RSD ^1^
	C	HI50	HI100		
No.	15	15	15		
Weight, g	368	370	381	0.5001	8.64
pH	6.24	6.32	6.30	0.1483	0.03
Thawing loss, %	0.97	0.96	0.95	0.9950	0.08
Proximate composition					
Water, %	76.4	76.0	76.1	0.5166	0.26
Protein, %	18.4	18.7	18.3	0.5636	0.28
Lipids, %	4.02	4.01	4.30	0.4379	0.18
Ash, %	1.18	1.23	1.28	0.1300	0.03
No.	6	6	6		
Cholesterol; mg/100 g meat	62.0 ^A^	59.4 ^B^	66.9 ^A^	0.0003	1.44
TBARs ^2^, mg MDA ^3^/kg meat	0.019	0.021	0.025	0.8216	0.01

^1^ Residual standard deviation; ^2^ Thiobarbituric acid reactive substances; ^3^ MDA = malondialdehyde; ^A, B^ Values within a row with different superscripts differ significantly (*p* < 0.01).

**Table 3:** Physico-chemical quality of the leg meat of chickens fed with 0% (C), 50% (HI50) and 100% (HI100) substitution of soybean oil with *Hermetia illucens* (HI) larvae fat in their finisher diets.

Physico-Chemical Traits	Experimental Groups			*p*-Value	RSD ^1^
	C	HI50	HI100		
No.	15	15	15		
Weight, g	391	403	413	0.0785	0.68
Thawing loss, %	0.42	0.32	0.39	0.4231	0.08
Cooking loss, %	3.95	3.60	3.45	0.5511	0.33
Total loss, %	4.37	3.92	3.84	0.4783	0.33
pH	6.39	6.41	6.41	0.8633	0.03
L*	47.1	46.8	48.1	0.3814	0.70
a*	2.21	2.60	2.33	0.8036	0.43
b*	12.9	14.3	14.5	0.0653	0.56

^1^ Residual standard deviation; L*: lightness; a*: redness; b*: yellowness.

**Table 4:** Chemical quality of the leg meat of chickens fed with 0% (C), 50% (HI50) and 100% (HI100) substitution of soybean oil with *Hermetia illucens* (HI) larvae fat in their finisher diets.

Chemical Traits	Experimental Groups			*p*-Value	RSD ^1^
	C	HI50	HI100		
No.	15	15	15		
Water, %	75.8	75.7	75.8	0.9017	0.19
Protein, %	16.8	17.0	16.7	0.7762	0.26
Lipids, %	6.31	6.18	6.31	0.9435	0.30
Ash, %	1.13	1.13	1.13	0.9603	0.02
No.	6	6	6		
Cholesterol, mg/100 g meat	80.2	79.7	81.5	0.3258	4.47
TBARs ^2^, mg/MDA ^3^ kg meat	0.068	0.073	0.061	0.7104	0.01

^1^ Residual standard deviation; ^2^ Thiobarbituric acid reactive substances; ^3^ MDA = malondialdehyde.

**Table 5:** Fatty acids profile (% of total fatty acid methyl esters; FAME) of the breast meat of chickens fed with 0% (C), 50% (HI50) and 100% (HI100) substitution of soybean oil with *Hermetia illucens* (HI) larvae fat in their finisher diets.

Fatty Acids	Experimental Groups			*p*-Value	RSD ^1^
	C	HI50	HI100		
No.	6	6	6		
C8:0	0.06 ^b^	0.07 ^a^	0.07 ^a^	0.0336	0.01
C10:0	0.04 ^C^	0.11 ^B^	0.18 ^A^	<0.0001	0.01
C12:0	0.01 ^C^	4.61 ^B^	11.0 ^A^	<0.0001	0.76
C14:0	0.32 ^C^	2.24 ^B^	4.63 ^A^	<0.0001	0.28
C15:0	0.08 ^C^	0.10 ^B^	0.12 ^A^	<0.0001	0.01
C16:0	18.1 ^C^	19.6 ^B^	20.4 ^A^	0.0004	0.77
C17:0	0.18 ^a^	0.18 ^a^	0.15 ^b^	0.0462	0.02
C18:0	9.23	8.91	8.80	0.4246	0.57
C20:0	0.21 ^A^	0.19 ^A,B^	0.13 ^B^	0.0002	0.02
C22:0	0.24	0.24	0.09	0.0911	0.13
C24:0	0.26	0.19	0.18	0.0888	0.07
Total SFA	28.8 ^C^	36.5 ^B^	45.8 ^A^	<0.0001	1.14
C14:1	0.03 ^C^	0.19 ^B^	0.40 ^A^	<0.0001	0.06
C15:1	0.09	0.11	0.11	0.5855	0.03
C16:1	1.31 ^C^	1.91 ^B^	2.69 ^A^	<0.0001	0.38
C17:1	0.05	0.06	0.06	0.2857	0.01
C18:1 *n*-9	23.9 ^A^	21.0 ^A,B^	18.4 ^B^	<0.0001	1.10
C20:1 *n*-9	0.30 ^A^	0.30 ^A^	0.21 ^B^	0.0002	0.03
C22:1 *n*-9	0.15	0.18	0.11	0.0664	0.09
Total MUFA	27.7 ^A^	25.6 ^B^	23.5 ^C^	0.0012	1.54
C18:2 *n*-6	30.0 ^A^	23.9 ^B^	17.6 ^C^	<0.0001	1.91
C18:3 *n*-6	0.13 ^C^	0.16 ^B^	0.20 ^A^	<0.0001	0.02
C20:2 *n*-6	0.60 ^A^	0.61 ^A^	0.48 ^B^	0.0028	0.06
C20:3 *n*-6	0.50	0.56	0.57	0.2824	0.07
C20:4 *n*-6	3.30	3.71	3.49	0.0578	0.27
C18:3 *n*-3	1.85 ^A^	1.27 ^B^	0.72 ^C^	<0.0001	0.18
C20:3 *n*-3	0.07	0.07	0.04	0.0686	0.02
C20:5 *n*-3	0.10	0.12	0.12	0.8432	0.06
C22:6 *n*-3	0.09 ^a^	0.08 ^a,b^	0.07 ^b^	0.0236	0.53
Total PUFA	36.8 ^A^	30.6 ^B^	23.4 ^C^	<0.0001	1.90
*n*-6	34.6 ^A^	29.0 ^B^	22.4 ^C^	<0.0001	1.75
*n*-3	2.19 ^A^	1.62 ^B^	1.03 ^C^	<0.0001	0.18
*n*-6/*n*-3	15.8 ^C^	18.0 ^B^	21.7 ^A^	<0.0001	1.24
AI	0.30 ^C^	0.59 ^B^	1.07 ^A^	<0.0001	0.04
TI	0.73 ^C^	0.96 ^B^	1.30 ^A^	<0.0001	0.05
PI	57.3 ^A^	51.3 ^B^	42.4 ^C^	<0.0001	1.50
HH ratio	2.03 ^A^	1.42 ^B^	0.95 ^C^	<0.0001	0.15

^1^ Residual standard deviation; SFA = saturated fatty acids; MUFA = monounsaturated fatty acids; PUFA = polyunsaturated fatty acids; UFA = unsaturated fatty acids; AI = Atherogenicity index; TI = Thrombogenicity index; PI = Peroxidability index; HH ratio = hypocholesterolemic/hypercholesterolemic ratio; ^a,b,c; A,B,C^ Values within a row with different superscripts differ significantly at *p* < 0.05; *p* < 0.001.

**Table 6:** Fatty acids profile (% of total FAME) of the leg meat of chickens fed with 0% (C), 50% (HI50) and 100% (HI100) substitution of soybean oil with *Hermetia illucens* (HI) larvae fat in their finisher diets.

Fatty Acids	Experimental Groups			*p*-Value	RSD ^1^
	C	HI50	HI100		
No.	6	6	6		
C8:0	0.05 ^C^	0.06 ^B^	0.07 ^A^	<0.0001	0.00
C10:0	0.04 ^C^	0.12 ^B^	0.21 ^A^	<0.0001	0.01
C12:0	0.06 ^C^	5.97 ^B^	13.2 ^A^	<0.0001	0.70
C14:0	0.35 ^C^	2.46 ^B^	4.73 ^A^	<0.0001	0.22
C15:0	0.08 ^B^	0.09 ^A,B^	0.11 ^A^	<0.0001	0.01
C16:0	16.9 ^B^	18.4 ^A,B^	18.7 ^A^	0.0061	0.86
C17:0	0.16 ^A^	0.13 ^B^	0.12 ^B^	0.0097	0.02
C18.0	7.76 ^a^	7.51 ^a,b^	6.76 ^b^	0.0279	0.60
C20:0	0.14 ^A^	0.13 ^A,B^	0.12 ^B^	0.0077	0.01
C22:0	0.10	0.04	0.07	0.1318	0.05
C24:0	0.19 ^A^	0.15 ^B^	0.11 ^C^	0.0055	0.04
Total SFA	25.8 ^C^	35.1 ^B^	44.2 ^A^	<0.0001	1.02
C14:1	0.06 ^C^	0.26 ^B^	0.52 ^A^	<0.0001	0.08
C15:1	0.04	0.05	0.03	0.1192	0.01
C16:1	2.17 ^C^	2.92 ^B^	3.68 ^A^	0.0024	0.61
C17:1	0.05 ^B^	0.04 ^B^	0.08 ^A^	0.0019	0.01
C18:1 *n*-9	24.7 ^A^	22.4 ^B^	20.2 ^C^	0.0003	1.41
C20:1 *n*-9	0.24	0.25	0.22	0.0875	0.02
Total MUFA	28.9	27.5	26.1	0.0991	2.07
C18:2 *n*-6	33.5 ^A^	26.2 ^B^	19.9 ^C^	<0.0001	1.80
C18:3 *n*-6	0.18	0.18	0.17	0.9298	0.02
C20:2 *n*-6	0.43 ^A^	0.47 ^A^	0.37 ^B^	0.0026	0.03
C20:3 *n*-6	0.37	0.40	0.37	0.1522	0.03
C20:4 *n*-6	2.85 ^A^	2.65 ^A,B^	2.27 ^B^	0.0186	0.32
C18:3 *n*-3	2.30 ^A^	1.57 ^B^	0.92 ^C^	<0.0001	0.14
C20:3 *n*-3	0.04 ^A^	0.05 ^A^	0.03 ^B^	0.0050	0.01
C20:5 *n*-3	0.09	0.16	0.10	0.0668	0.05
C22:6 *n*-3	0.07	0.06	0.06	0.8159	0.01
Total PUFA	39.9 ^A^	31.8 ^B^	24.3 ^C^	<0.0001	2.06
*n*-6	37.3 ^A^	29.9 ^B^	23.1 ^C^	<0.0001	1.94
*n*-3	2.59 ^A^	1.93 ^B^	1.20 ^C^	<0.0001	0.15
*n*-6/*n*-3	14.4 ^C^	15.5 ^B^	19.2 ^A^	<0.0001	0.91
AI	0.27 ^C^	0.58 ^B^	1.01 ^A^	<0.0001	0.04
TI	0.61 ^C^	0.82 ^B^	1.07 ^A^	<0.0001	0.05
PI	59.5 ^A^	50.1 ^B^	40.0 ^C^	<0.0001	2.32
HH ratio	2.35 ^A^	1.55 ^B^	1.05 ^C^	<0.0001	0.17

^1^ Residual standard deviation; SFA = saturated fatty acids; MUFA = monounsaturated fatty acids; PUFA = polyunsaturated fatty acids; UFA = unsaturated fatty acids; AI = Atherogenicity index; TI = Thrombogenicity index; PI = Peroxidability index; HH ratio = hypocholesterolemic/hypercholesterolemic ratio; ^a,b,c; A,B,C^ Values within a row with different superscripts differ significantly at *p* < 0.05; *p* < 0.001.

**Table 7:** Sensory traits of the breast meat of chickens fed with 0% (C), 50% (HI50) and 100% (HI100) substitution of soybean oil with *Hermetia illucens* (HI) larvae fat in their finisher diets.

Descriptors	Experimental Groups			*p*-Value	RSD ^1^
	C	HI50	HI100		
No.	15	15	15		
Chicken odor	5.56	5.83	5.51	0.6354	0.91
Chicken flavor	5.63	5.57	5.35	0.5779	1.11
Saltiness	4.88	4.83	4.83	0.9293	0.15
Acidity	2.74	2.63	2.69	0.9944	0.01
Unctuosity	4.69	4.64	4.54	0.9264	0.16
Juiciness	4.70	4.98	4.58	0.4497	1.59
Fibrousness	5.84	5.98	6.32	0.4073	1.84
Chewiness	5.24	5.61	5.19	0.4150	1.80
Adhesiveness	5.17	5.36	5.13	0.6494	0.86
Toughness	5.70	5.45	5.68	0.9526	0.11

^1^ Residual standard deviation.
